# A case of advanced intrahepatic cholangiocarcinoma accidentally, but successfully, treated with capecitabine plus oxaliplatin (CAPOX) therapy combined with bevacizumab: a case report

**DOI:** 10.1186/s40792-016-0191-0

**Published:** 2016-06-25

**Authors:** Masahito Uji, Takashi Mizuno, Tomoki Ebata, Gen Sugawara, Tsuyoshi Igami, Keisuke Uehara, Masato Nagino

**Affiliations:** Division of Surgical Oncology, Department of Surgery, Nagoya University Graduate School of Medicine, 65 Tsurumai-cho, Showa-ku Nagoya, 466-8550 Japan

## Abstract

Although surgical resection is the only way to cure biliary tract cancer (BTC), most BTCs are unresectable by the time they are diagnosed. Chemotherapy is usually used to treat unresectable BTC, but its impact on survival is small. Here, we report the case of a 70-year-old woman with a locally advanced intrahepatic cholangiocarcinoma that was initially diagnosed as an unresectable liver metastasis from colon cancer that had invaded all of the major hepatic veins. However, the tumor was noticeably reduced after treatment with CAPOX plus bevacizumab, which is an uncommon therapy for BTC. The tumor was finally resected by inferior right hepatic vein-preserving left hepatic trisectionectomy combined with a resection of the right hepatic vein after a right hepatic vein embolization.

## Background

Biliary tract cancer (BTC), which includes multiple cancers, such as cholangiocarcinoma and gallbladder carcinoma, is difficult to treat. Although surgical resection is the only way to cure, most patients with BTC are diagnosed at such a late stage that resection is not possible. Systemic chemotherapy is used for these patients [1, 2], and recently, combination therapy with gemcitabine and cisplatin (GC) has been recommended as the first-line regimen in these cases [3, 4]. A large phase III trial conducted in the UK added cisplatin to a gemcitabine therapy regimen and demonstrated a subsequent increase in response rate (26 vs 16 %) and increase in overall survival time (11.7 vs 8.1 months) [3]. Other regimens of chemotherapy were assessed in several clinical trials, but the findings are inconsistent because of the limited number of patients who enrolled in the trials.

We herein report a case of intrahepatic cholangiocarcinoma that was accidentally, but successfully, treated with a new chemotherapy regimen that was not previously used to treat BTC.

## Case presentation

Liver dysfunction was discovered in a 70-year-old woman at a local hospital. Abdominal ultrasonography revealed a hepatic mass, and she was referred to our hospital for possible surgery. On admission, laboratory tests showed mild elevations of AST, ALT, and γGTP. Serum levels of CEA and CA19-9 were 5.8 ng/L (normal range 0–5 ng/L) and 243 IU/mL (normal range 0–37 IU/mL), respectively.

**Fig. 1:**
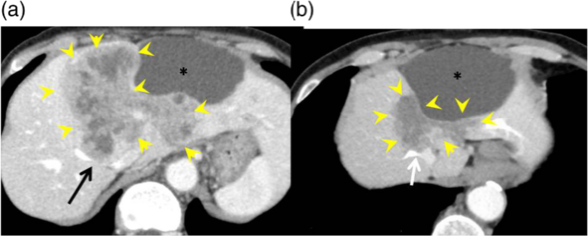
Multidetector-row computed tomography. **a** A large hepatic tumor occupying the left lobe and the right anterior sector of the liver (*yellow arrowheads*). The left and middle hepatic veins were occluded by invasion of the tumor, and the tumor also invaded the right hepatic vein (*black arrow*). **b** The liver tumor substantially decreased in size after chemotherapy (*yellow arrowheads*); however, it was still invading all of the major hepatic veins (*white arrow*). An asterisk indicates the liver cyst

Multidetector-row computed tomography (MDCT) showed a large hepatic tumor, 96 × 80 mm in diameter, occupying the left lobe and right anterior sector of the liver. The left and middle hepatic veins were occluded by tumor invasion, and the tumor had also invaded the right hepatic vein (RHV) near where it enters the inferior vena cava (IVC) (Fig. 1a). A colonoscopy was performed as a screening test, and it showed a type 2 tumor in the ascending colon. Pathological analysis of the colon tumor determined that it was a well-differentiated adenocarcinoma. The diagnosis of colon cancer with a liver metastasis was made. The liver metastasis was solitary but unresectable because of invasion into all of the major hepatic veins.

**Fig. 2:**
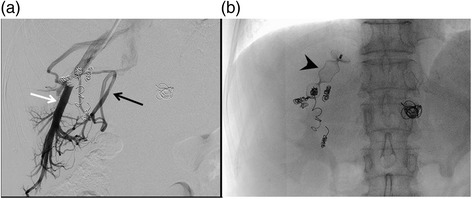
**a** Retrograde venography of the right hepatic vein under balloon occlusion. The right hepatic vein was large (*white arrow*), and the inferior right hepatic vein was thin (*black arrow*). **b** The right hepatic vein was embolized with an Amplatzer Vascular Plug® (*black arrowhead*). The other steel coils were used for portal vein embolization

**Fig. 3:**
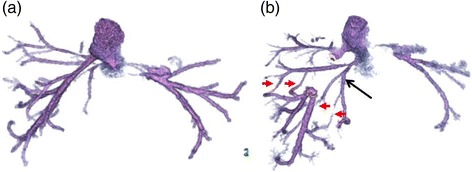
**a** Three-dimensional CT venography prior to right hepatic vein embolization. **b** Three-dimensional CT venography after right hepatic vein embolization. Several collaterals from the right hepatic vein to the inferior right hepatic vein (*black arrow*) were observed (*red arrows*)

We decided to treat this patient with a chemotherapy regimen of capecitabine plus oxaliplatin (CAPOX) combined with bevacizumab. After nine courses of the regimen, the size of the liver tumor was substantially decreased to 50 × 35 mm in diameter (Fig. 1b), but it was still invading all of the major hepatic veins. Nevertheless, we judged that the tumor could be resected by left hepatic trisectionectomy with RHV resection, due to the presence of the inferior right hepatic vein (IRHV). For a safe hepatectomy, embolization of the left and right anterior portal veins was performed. Seven days after this embolization, the RHV was also embolized to develop collaterals from the RHV to the IRHV and also to avoid RHV reconstruction (Fig. 2). MDCT, performed 14 days after the RHV embolization, demonstrated that venous perfusion to the IRHV was increased and that the volume of the right posterior sector was 496 cm^3^ (51.2 % of the whole liver) (Fig. 3). The plasma disappearance rate (K) of indocyanine green (ICG) was 0.153, so the future remnant ICG K was calculated to be 0.078.

**Fig. 4:**
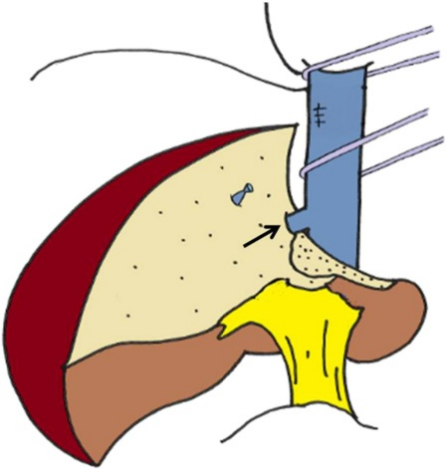
Scheme of the performed surgery (left trisegmentectomy with resection of the right hepatic vein). The *black arrow* indicates the preserved inferior right hepatic vein

Surgery for the hepatic tumor was performed 18 days after embolization of the RHV. A left hepatic trisectionectomy was performed. The distal part of the RHV and the IVC near the entry of the RHV were involved and were resected en bloc. The defect in the IVC wall was sealed with direct closure (Fig. 4). The operation time was 443 min, and the blood loss was 2406 mL. Fifty-three days after the hepatectomy, a right hemicolectomy was performed. The patient was discharged from the hospital 79 days after the hepatectomy in good health.

**Fig. 5:**
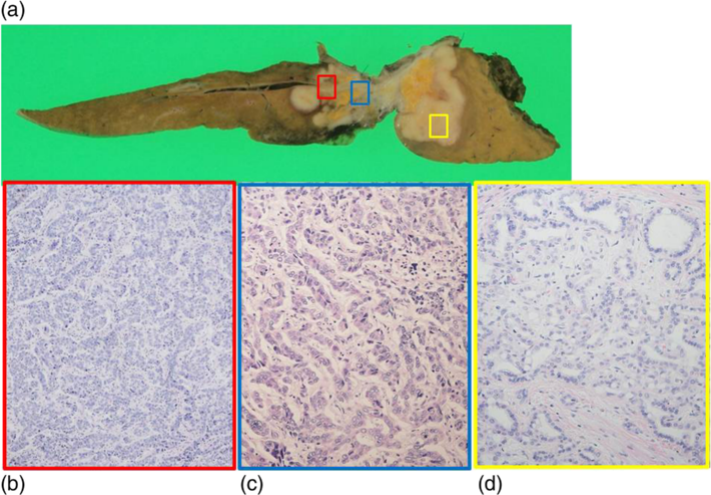
**a** Cut surface of the resected specimen. **b**–**d** Microscopic findings of the liver tumor. The tumor cells that proliferated in an alveolar (**b** *red frame*) and a thin-trabecular pattern (**c** *blue frame*) and showed a tubular formation (**d** *yellow frame*)

**Fig. 6:**
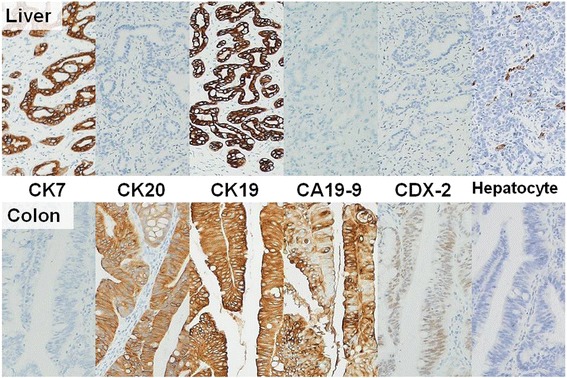
Immunohistochemical findings. Immunohistochemical findings differed between the liver tumor and the cancerous colon tissue. Liver: CK7(+), CK20(−), CK19(+), CA19-9(−), CDX-2(−), hepatocyte(−); colon: CK7(−), CK20(+), CK19(+), CA19-9(+), CDX-2(+), hepatocyte(−)

Histologically, the cancerous colon tissue was moderately differentiated adenocarcinoma. The liver tumor did not have a fibrous capsule with invasive growth into the liver parenchyma. The tumor cells proliferated with an alveolar and thin-trabecular pattern (Fig. 5). Immunohistochemically, the liver tumor was positive for CK7 and CK19 and negative for CK20, CA19-9, and CDX-2. In contrast, the cancerous colon tissue was positive for CK19, CK20, CA19-9, and CDX-2 and negative for CK7. Hepatocytes were negative in both the liver and colon tumors (Fig. 6). Based on this finding, we determined that the liver tumor was not a metastasis from the colon cancer, but was a primary intrahepatic cholangiocarcinoma.

Several clinical studies have assessed the clinical efficacy of various chemotherapy treatment regimens in patients with advanced BTC, but none of the studies evaluated CAPOX therapy combined with bevacizumab. Only one phase II trial assessed CAPOX therapy for BTC, which includes gallbladder carcinoma and extra- and intrahepatic cholangiocarcinomas, and the trial showed that the overall response rate (CR, PR, or SD) was 65 % [5]. However, in patients with an intrahepatic cholangiocarcinoma, neither CR nor PR was achieved, and SD was observed in only 33 % of the patients, with a median overall survival time of 5.2 months. Thus, this phase II trial suggested that CAPOX therapy has no effect on intrahepatic cholangiocarcinoma.

Bevacizumab is an important therapeutic agent with promising results against some malignancies, and it is frequently given in conjunction with CAPOX therapy. The ability of this molecularly targeted drug, in combination with gemcitabine plus oxaliplatin (Gemox) or with erlotinib, to treat BTC was tested in two phase II trials [6–9]. The combination of Gemox plus bevacizumab showed promising results with a response rate of 40 % and a median overall survival time of 12.7 months [8]. In patients with intrahepatic cholangiocarcinoma, the median overall survival time was 14.2 months. In contrast, bevacizumab plus erlotinib showed modest efficacy with a response rate of 12 % and a median overall survival of 9.9 months [9]. Although the effect of CAPOX plus bevacizumab therapy for intrahepatic cholangiocarcinoma on survival is unclear, this combination regimen was very effective in the current case report. Vascular endothelial growth factor (VEGF) is expressed in approximately 50 % of intrahepatic cholangiocarcinomas [10], and anti-VEGF treatments result in pruning of the tumor vasculature and reductions in vessel tortuosity [11]. MDCT images showed that the current patient had a hypervascular liver tumor. Microscopically, scattered unpaired arteries were found via immunostaining for heavy-caldesmon, a marker of vascular smooth muscle. These histological findings indicate that bevacizumab might be an effective treatment for intrahepatic cholangiocarcinoma.

In 1987, Makuuchi et al. reported the possibility of using IRHV-preserving left hepatic trisectionectomy with combined resection of the RHV [12]. Thereafter, this challenging extended hepatectomy was performed in a few cases where the IRHV was “thick” [13, 14]. In cases where the IRHV is “thin,” however, this surgery can be risky; therefore, to overcome the risks, the use of RHV embolization was suggested by Nagino et al. [15]. They proposed that preoperative RHV embolization be used when the RHV was large and the IRHV was medium or small [16]. The IRHV in the patient in the current case was thin, approximately 0.5 cm in diameter and, therefore, was a good candidate for RHV embolization.

## Conclusions

This was a rare case of advanced intrahepatic cholangiocarcinoma that was accidentally, but successfully, treated with a CAPOX plus bevacizumab regimen. The current case could provide insight for new chemotherapy regimen options to treat BTC.

## Consent

Written informed consent was obtained from the patient for publication of this case report and any accompanying images. A copy of the written consent is available for review by the Editor-in Chief of this journal.

## Abbreviations

BTC, biliary tract cancer; CAPOX, capecitabine plus oxialiplatin; CR, complete response; GC, gemcitabine plus oxaliplatin; GEMOX, gemcitabine plus oxaliplatin; IRHV, inferior right hepatic vein; IVC, inferior vena cava; LHV, left hepatic vein; MDCT, multidetector-row computed tomography; MHV, middle hepatic vein; PR, partial response; RHV, right hepatic vein; SD, stable disease; VEGF, vascular endothelial growth factor.
